# Machine learning prediction of oncology drug targets based on protein and network properties

**DOI:** 10.1186/s12859-020-3442-9

**Published:** 2020-03-14

**Authors:** Zoltán Dezső, Michele Ceccarelli

**Affiliations:** 1 Computational Biology-Genomic Research Center, ABBVIE, Redwood City, CA USA; 2 Department of Electrical Engineering and Information Technology (DIETI), University of Naples “Federico II”, 80128 Naples, Italy; 3 Istituto di Ricerche Genetiche “G. Salvatore”, Biogem s.c.ar.l, 83031 Ariano Irpino, Italy

**Keywords:** Machine learning, Drug target prioritization, Network analysis

## Abstract

**Background:**

The selection and prioritization of drug targets is a central problem in drug discovery. Computational approaches can leverage the growing number of large-scale human genomics and proteomics data to make in-silico target identification, reducing the cost and the time needed.

**Results:**

We developed a machine learning approach to score proteins to generate a druggability score of novel targets. In our model we incorporated 70 protein features which included properties derived from the sequence, features characterizing protein functions as well as network properties derived from the protein-protein interaction network. The advantage of this approach is that it is unbiased and even less studied proteins with limited information about their function can score well as most of the features are independent of the accumulated literature. We build models on a training set which consist of targets with approved drugs and a negative set of non-drug targets. The machine learning techniques help to identify the most important combination of features differentiating validated targets from non-targets. We validated our predictions on an independent set of clinical trial drug targets, achieving a high accuracy characterized by an Area Under the Curve (AUC) of 0.89. Our most predictive features included biological function of proteins, network centrality measures, protein essentiality, tissue specificity, localization and solvent accessibility. Our predictions, based on a small set of 102 validated oncology targets, recovered the majority of known drug targets and identifies a novel set of proteins as drug target candidates.

**Conclusions:**

We developed a machine learning approach to prioritize proteins according to their similarity to approved drug targets. We have shown that the method proposed is highly predictive on a validation dataset consisting of 277 targets of clinical trial drug confirming that our computational approach is an efficient and cost-effective tool for drug target discovery and prioritization. Our predictions were based on oncology targets and cancer relevant biological functions, resulting in significantly higher scores for targets of oncology clinical trial drugs compared to the scores of targets of trial drugs for other indications. Our approach can be used to make indication specific drug-target prediction by combining generic druggability features with indication specific biological functions.

## Background

Drug target identification is one of the most critical steps in the pre-clinical drug development pipeline and the choice of the right target can significantly impact the chances of advancing the drug to clinical development and the success of the clinical trials. With the accumulation of approved and clinical trial drugs, it has become clear that successful drug targets share several important features, which include having a disease relevant biological function and certain properties that would favor the existence of binding sites, thus making the protein capable of binding to small molecules.

There are a variety of experimental techniques for target identification including affinity pull downs, pooled RNAi data [1] and more recently genome-scale CRISPR–Cas9 screens [2]; however, these methods are expensive and labor intensive and not without limitations. Computational approaches on the other hand can leverage the growing number of large-scale human genomics and proteomics data sets to make *in-silico* target identification, thus potentially reducing substantially the cost and the time needed to assess a target. A targeted computational approaches for example is the structure-based drug discovery where protein druggability is determined through molecular docking methods which can predict binding sites and binding affinity of the target proteins [3]. These methods however are limited in finding novel targets because the three-dimensional structure of most proteins is not readily available.

Several databases aim to systematically capture the expanding number of approved and clinical trial drugs, including their indications and corresponding targets. Currently the DrugBank database [4, 5] contains 2594 approved small molecule drugs and 1289 approved biotech drugs. Another comprehensive collection of drug information, the Therapeutic Target Database (TTD) [6], contains 2544 drugs and 2589 corresponding targets, as well as drug resistance mutations and gene expression profiles after treatments for some of the drugs. The existence of such databases allows us to find common characteristics among the successful drug targets and to use these features in combination with other knowledge to guide novel drug discovery research.

Many data-driven approaches have focused on gene expression changes after drug treatment to predict similarity between drugs and potentially predict shared targets [7–9]. Several studies combined different data types to improve the prediction of drug similarity [10] and to predict drug-target interactions [11, 12]. Other methods focused on predicting the probability of success in a clinical trial by estimating the toxicity based on several chemical properties, drug-likeness measures of the molecules and the target properties [13].

Computational approaches to identify novel targets are often limited by the availability of data for less studied proteins. We propose an unbiased approach whereby novel target identification leverages the characterization of all proteins based on properties that are known or predicted based on protein sequence or genome-wide experimental data. Machine learning techniques provide the opportunity to identify the most important combination of features differentiating validated targets from non-targets. In this approach, the first step is to build a model on a training set which consists of targets with approved drugs and a negative set of non-drug targets. The model can be used to generate a druggability score of the potential novel targets. A similar approach was first used by Bakheet and Doig [14, 15] and followed by several other works where the performance of the models was improved by adding new features [16]. The protein features in these models included properties derived from the sequence and other protein functions such as gene ontology, essentiality based on mouse gene knockdown experiments and tissue specificity. The advantage of this approach is that is it unbiased and can be used to evaluate proteins with limited information about biological function or essentiality using a variety of properties determined from the amino acid sequence of the protein.

Here we hypothesized that druggability of a target can be indirectly and automatically derived from a set of proteins that have been successfully identified as good drug targets (positive set) with a machine learning approach that discovers their shared properties compared to a negative set of proteins. The small set of known validated drug targets and the large set of unknown potential targets resulted in an unbalanced positive-only learning task [17, 18], requiring us to base our approach on bagging thousands of Random Forest classifiers trained on different instances of the negative set to achieve high accuracy predictions in identifying the trial drugs in clinical trials not belonging to the training set.

Network features have been shown to be particularly important to score potential drug target proteins [19, 20] There are several differences between our approach and the work presented in [19, 20], first, we focus just on oncology drug target and use as additional functional feature an ad hoc representation of the pathways where each candidate protein is involved, second we show that just five features encoding the protein network properties are enough to efficiently score the drug target with high accuracy, and finally we adopt a bagging approach to take into account the lack of appropriate negative examples of oncology drug targets. We also report the application of our approach to the whole drugbank dataset as was performed in [19, 20].

We focused our predictions on oncological targets by scoring proteins by the most cancer-relevant biological processes, molecular functions and signaling pathways based on a manually curated database [21].

The proposed approach can be readily used to make indication-specific drug-target prediction by combining generic druggability features with indication-specific biological functions.

## Results

### Properties of drug targets

We determined the set of approved oncology drugs based on the current version of the Therapeutic Target Database [6]. Approved, clinical and investigational drugs with corresponding indication and their respective targets were downloaded from the database. The data was filtered and curated to determine a high confidence list of approved and clinical trial oncology targets. Our final list consisted of 102 targets for approved drugs and an additional 277 targets for clinical drugs (Table S1).

Next, we determined a comprehensive list of 70 properties for all human proteins by combining the manually curated literature captured in the Swiss-prot database, the computational predictions for missing features and network centrality properties calculated based on the protein-protein interaction network (Methods). The set of features included standard characteristics based on the protein sequences from Swiss-prot database [22] (such as molecular weight and physicochemical classes) as well as several other properties that have been shown to differentiate drug-targets from non-drug targets based on previous publications [14–16]. These features included: subcellular localization, post-translational modifications, enzyme classification, PEST region (peptide sequence enriched in proline, glutamic acid, serine and threonine amino acids), secondary structure, signal peptide cleavage, protein essentiality, solvent accessibility, and tissue specificity (for a full list of features see Table S2). Protein essentiality was determined based on mouse homozygous loss-of function mutations that lead to lethality and mapped through orthologs to human proteins [23].

In addition, we calculated network centrality measures for each protein based on the protein-protein network information from the STRING database [24]. It has been shown that network properties of proteins correlate with their biological functions, essentiality and tissue specificity [25, 26]. Therefore, the network information complements these other properties further helping with the evaluation of less studied proteins where information about biological function and essentiality may be limited.

To capture the known biological functions of the proteins we used the comprehensive ontology database of the commercially available database of Metacore [21] and scored the proteins based on biological processes, molecular function, and signaling pathways. The scoring of the protein biological functions was done according to the ranked ontologies ordered by the enrichment analysis of the targets in the training set (Methods). This procedure captured the most significant gene ontology categories for the validated oncology targets in the positive training dataset.

**Fig. 1:**
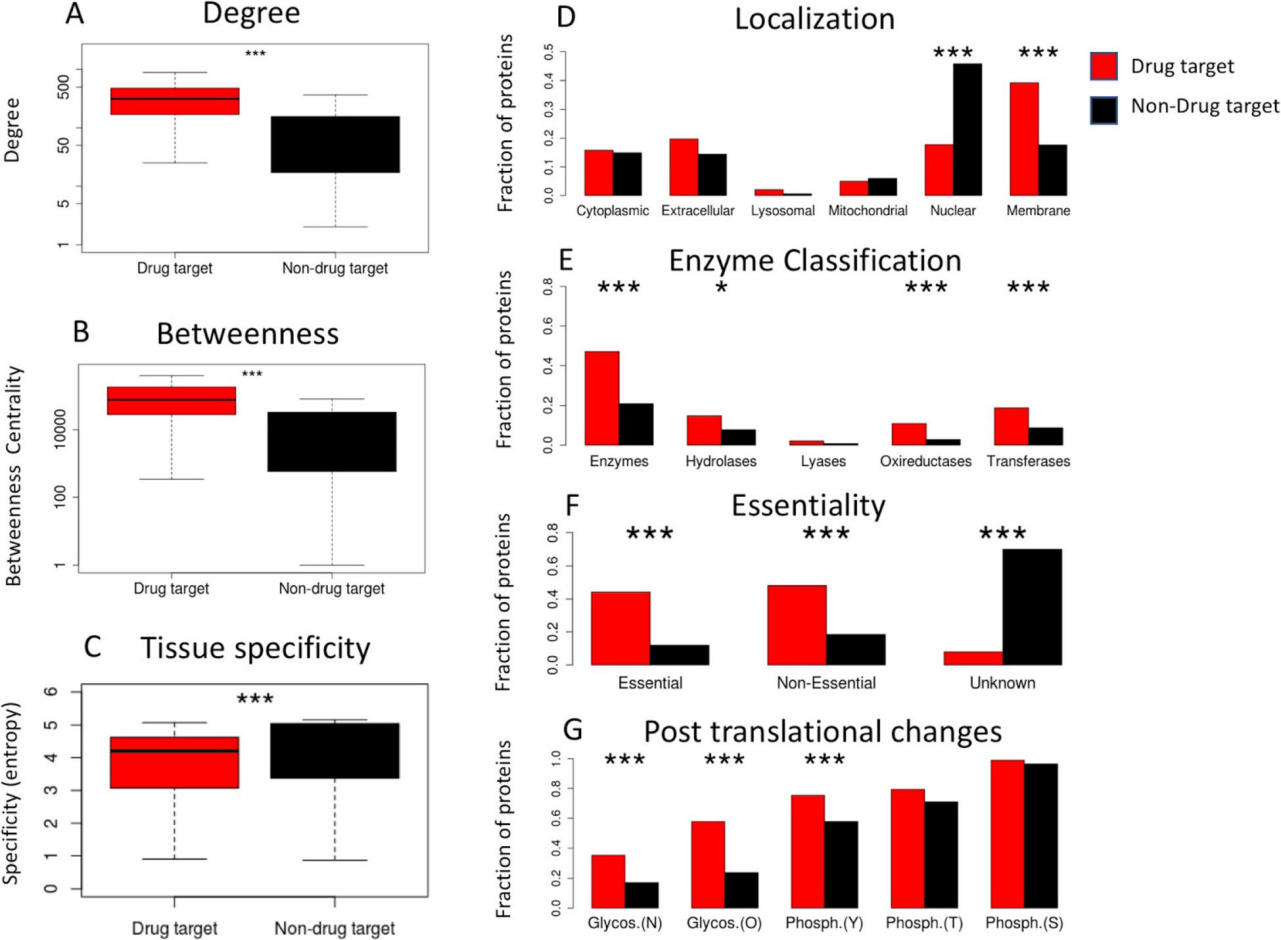
Top features differentiating drug targets from non-targets. The features are significantly different between drug targets and non-targets. The asterisk marks the significance level of the difference: *p* < 0.05 indicated as *, *p* < 0.01 indicated as ** and *p* < 0.001 indicated as ***. The red and black bars correspond to drug and non-drug targets, respectively. **a** Degree or the number of interactions of the proteins. **b** Betweenness centrality of the proteins. **c** Tissue specificity of proteins characterized by Shannon entropy. **d** Subcellular localization of proteins. **e** Enzyme classification of proteins. **f** Essentiality of proteins. **g** Fraction of protein characterized by different post translational modifications

Next, we performed statistical tests to identify the feature which were significantly different between the set of drug targets and non-drug targets (Fig. 1). Our main findings were in good agreement with previous studies [14, 15]. For example, we found that targets were more likely to be membrane proteins (*p* = 2.33*10^− 7^), were enriched in enzymes (*p* = 2.8*10^− 11^) and tended to be tissue-specific (1.6*10^− 8^). We found the presence of more glycosylation sites (*p* = 9.8*10^− 20^) possibly indicating longer half-lives for drug targets. The target essentiality status, determined from mouse knock-out studies [23], was significantly more established compared to the non-targets (*p* < 2.2*10^− 16^) reflecting the fact that the drug targets are in general more studied proteins.

Interestingly, the network properties of the proteins showed large differences between drug- and non-drug targets (*p* < 2.2*10^− 16^ for all network measure). Indeed, it has been observed in previous studies that drug targets tend to have a higher number of connections compared to non-target proteins based on unbiased high-throughput yeast two-hybrid system [25]. We found that the commonly used network centrality measures showed some of the most significant differentiation between targets and non-targets (Fig. 1). We included a complete list of differentiating features in supplementary data (Table S2 and Figure S1).

### Machine learning prediction of target “druggability”

**Fig. 2:**
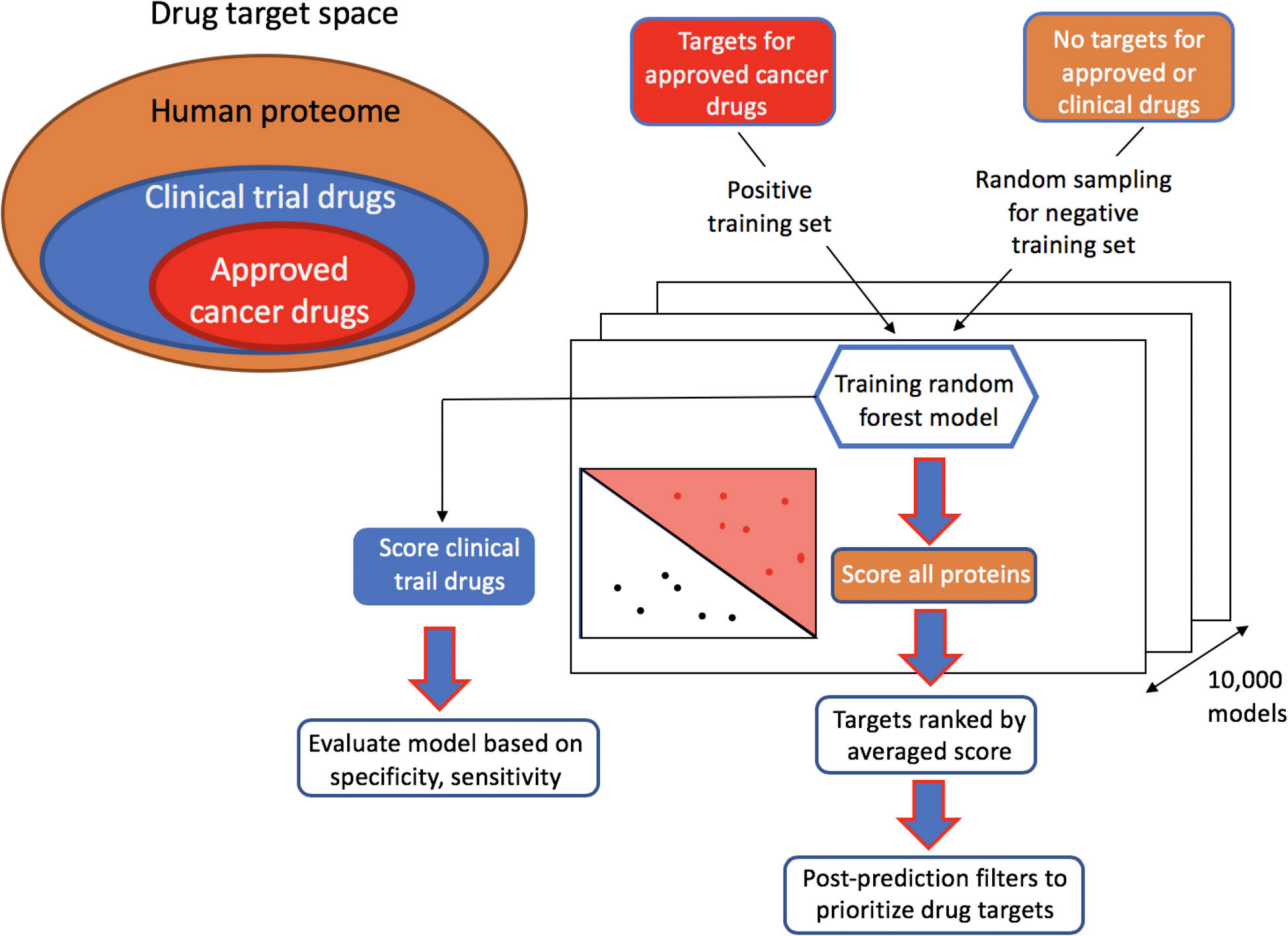
Overview of the drug target druggability predictions. Our positive training set consisted of 102 approved oncology drug targets. We generated negative training sets of the same size as the positive set by random sampling without replacement all human proteins after excluding both the approved and clinical trial oncology targets. We built a large number of 10,000 random forest models using each of the random negative sets and made predictions based on each model. We then assign a drug target probability score to each protein by averaging the predictions of the 10,000 models. To evaluate the performance of our model we considered the independent set of 277 targets which had at least one oncology clinical trial drug targeting them, but no approved drugs

**Fig. 3:**
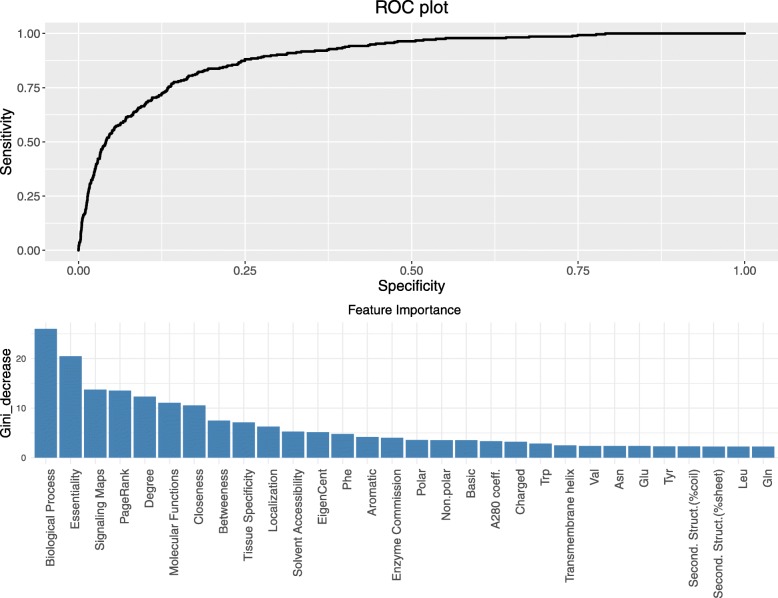
Prediction performance and the most predictive features. **a** Receiver operating characteristic (ROC) curve showing the performance of the model. The plot is characterized by an AUC (area under the curve) of 0.89. **b** The decrease in Gini for top features. The bar plot shows the features ordered by decreasing importance of prediction in the models

Our main objective is to use the set of 70 protein features described above to prioritize and score proteins in order to promote the discovery of novel targets and/or to filter the candidate list of targets. The set of 102 targets of approved cancer drugs is our positive training set, this is the typical setting where a learner only has access to positive examples and unlabeled data because in the set of proteins outside this small collection of approved targets there are many targets (for example in the pipelines) or targets still to be considered or discovered. This kind of problem is known in machine learning as *Positive Unlabeled* (PU) [17, 27] with the additional complication of the high unbalance [28] between the positive set and the wide set on unlabeled samples. Here we adopt an approach combining *easy ensemble* [28] and *bagging* [29] as shown in Fig. 2. In order to have a balanced training set for our model we generated negative training sets of the same size of the positive set by random sampling without replacement all human proteins after excluding both the approved and clinical trial oncology targets. We built 10,000 random forest models using each of the random negative sets and made predictions based on each model. We then assigned a drug target probability score to each protein by averaging the predictions over the 10,000 models. To evaluate the performance of our model we considered the independent set of 277 targets which had at least one oncology clinical trial drug targeting them, but no approved drugs. We achieved a high-accuracy prediction in identifying the clinical trial drugs resulting in an AUC of 0.89 (Fig. 3a and Table S3). Furthermore, we investigated if the threshold on the protein-protein interaction confidence would affect our results, but found no significant changes in prediction accuracy (Table S3A). We note that we tested neural network models as well for the predictions and the results and prediction accuracy was very similar to the random forest models (results not shown). The list of targets for approved cancer drugs used for the training set and the clinical trial drug targets for validation was included in the supplementary data (Table S1).

To further evaluate our predictions, we compared our drug target score with a recent genome-scale CRISPR screen by Behan et al. [2], where the authors defined a priority score based on a 324 cancer cell line panel. We found that our prediction score had a significant correlation with the priority score calculated from the CRISPR screen (*p* = 5.5*10^− 12^ significant correlation) validating our findings.

### Predictive features of target “druggability”

In our models, we included all features, regardless of how well they differentiated drug targets form non-drug targets. Most of the features included can be considered as independent variables, however, there were few subsets such as the network centrality measures or the gene ontology classifications which were strongly correlated (Fig. S2). Nevertheless, we found that the random forest models were able to pick the most important variables for the predictions. To characterize the relative importance of the protein features in the predictions we looked at the mean decrease of Gini metric [30]. We averaged the variable importance over 10,000 models giving us an overall estimate of feature importance (Fig. 3b). The top measures include the biological function of proteins, network centrality measures (page-rank, closeness, betweenness, degree), protein essentiality, tissue specificity, localization, and solvent accessibility. Indeed, it is expected that the biological function is a crucial feature of a drug target protein, because a protein may be druggable based on its 3D structure and other properties, but if it is not involved in disease-relevant processes targeting it with a drug will have minimal impact on the disease state.

The different network centrality measures and protein essentiality are correlated as shown in previous studies [31]. The combination of biological function, essentiality and centrality measures reflects the fact that the target not only has to have the right biological function, but the high centrality also assures that targeting it with a drug will have a high impact on those processes. Another important feature for our predictions was tissue specificity. Indeed, tissue-specific genes have been shown that are more likely to be drug targets [26, 32] due to the reduced risk of side effects.

**Fig. 4:**
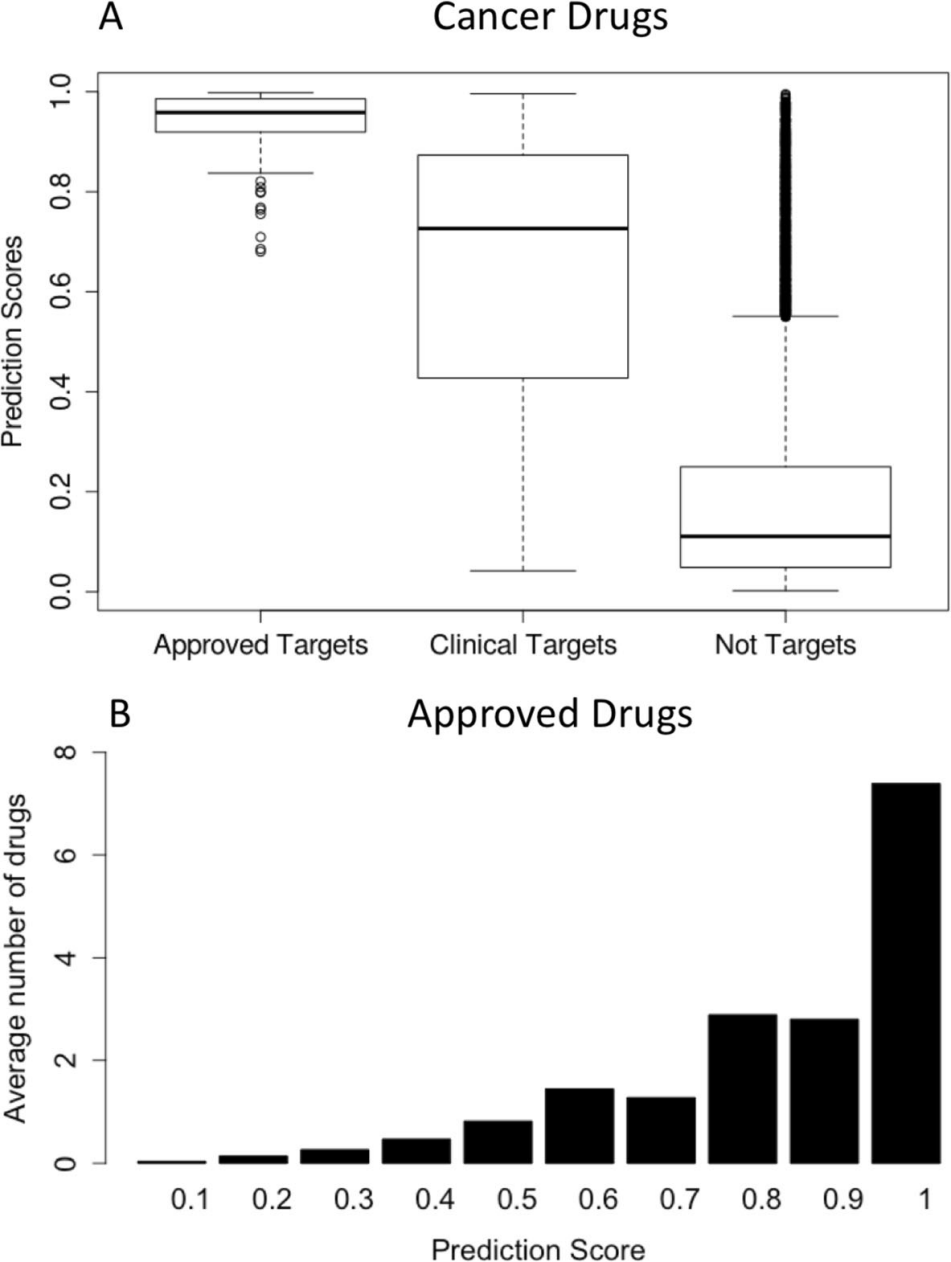
Predicting novel drug targets. **a** Predictions scores. We compared the distribution of predictions scores among the training set (approved cancer drug targets), validation set (clinical drug targets) and the rest of the proteins. The median score for the approved targets is the highest (0*.*96), followed by the clinical trial targets (0.73) and the rest of non-target proteins (0.11). The subset of proteins from the non-target set characterized with high scores are good novel drug target candidates. **b** Correlation of the number of currently approved drugs and the prediction scores of their corresponding targets

We compared our predictions scores for the approved oncology targets, clinical targets and the rest of the proteins (Fig. 4a). As expected, our training data had the highest score with a median of 0.96. Nevertheless, the independent set of cancer clinical targets was also characterized by a high median score of 0.73 compared to the rest of the non-target proteins which had a median score of only 0.11. It is expected that a large fraction of proteins is not good drug targets because of poor druggability, toxicity or disease irrelevant biological functions. However, the set of non-drug targets had a subset of 2117 outliers, proteins characterized with scores larger than 0.5 (Fig. 4a). We believe this subset of proteins may contain potentially interesting candidates for novel drug targets.

Next, we compared our predictions with existing drug information from the DrugBank (Fig. 4b). We found a strong positive correlation between the number of currently approved drugs and the prediction scores of their corresponding targets. Indeed, the proteins with scores of at least 0.7 had in average at least two drugs targeting them. Our top scored proteins (> 0.9) were well established targets, being targeted on average by nearly eight drugs (Fig. 4b). These results show that using only a small set of 102 validated targets we were able to recover the majority of known drug targets with high confidence. Although our predictions were based on a training set of oncology targets, many of the druggability features in our model most likely were not specific to oncology targets. However, because we scored higher the cancer specific biological functions, we expected that cancer targets may score higher compared to the targets of other indications. In order to test this, we compared the scores of 540 non-cancer clinical trial targets with the scores of the 277 cancer clinical trial targets from TTD. Indeed, we found a small but significant (*p* = 0.02) difference between the scores of non-cancer and cancer clinical trial targets (0.64 versus 0.73). Additionally, we downloaded a set of 402 targets of withdrawn drugs from the DrugBank. A small set of 20 targets of withdrawn drugs were identified as not having any approved drug targeting them. We found that the scores of targets associated only with withdrawn drugs were significantly smaller compared to the targets of drugs with ongoing clinical trials (0.37 vs 0.73, Figure S3). We note that the set of targets associated with withdrawn targets was rather small, as most drugs fail not necessarily because of their targets, but issues related to efficacy, off-target effects and clinical trial design.

Almost all the highest scored proteins not included in the training set had existing drugs targeting them (Table S4). The target proteins with highest scores included many well validated oncology targets (for example: EGFR, VEGF, c-KIT and c-Met).

The indication specificity of the predictions can be further enhanced by filtering the candidate targets using data specific for the indication of interest. For example, oncology targets could be further filtered down by correlation analysis of gene expression with overall survival in cancer based on public data such as the Cancer Genome Atlas (TCGA) [33]. Similarly for immune-oncology targets it is possible to focus on targets with immune cell specific expression profiles based on publicly available datasets such as for example Database of Immune Cell EQTLs (DICE) [34].

### Comparisons with similar approaches

We compared our prediction performance with other similar publications [15, 16]. Direct comparison between these approaches is difficult due to the differences in training and validation sets, the use of different features and the differences in the performance metrics reported. We would note that the main difference between our approach and the publications of Kim et al. and Bakheet at al [15, 16]. are in the addition of network centrality measures and our rank-based scoring of the oncology relevant biological functions. Indeed, including these features had a significant impact on improving the prediction accuracy (Table S3C) and also enabled us to prioritize targets based on our indication of interest. Our leave-one -out cross validation averaged over 100 random non-target sets achieved a sensitivity, specificity and accuracy around 0.91 and an AUC value of 0.97 (Table S3A). These values show improvement compared to the sensitivity between 0.64–0.85 reported by Kim et al. and the accuracy of 0.89 reported by Bakheet et al. Finally, the publication of Li et al. is similar in their choice to include several network properties. Direct comparison is difficult since they use a very large set of different features. Nevertheless, we performed a cross validation on the 1100 drug targets they utilized in their model and performed a cross validation using random sampling for the negative drug target set (Table S3B). Our performance on this set of targets was very similar within the sampling error to the prediction performance reported by Li et al. (sensitivity is 0.87 vs 0.9; specificity is 0.85 vs 0.8; accuracy 0.87 vs 0.85) Our approach has higher prediction performance when focusing only on oncology targets by applying a rank-ordered scoring method that can prioritize cancer specific biological functions and potentially can make predictions specific to any indication of interest.

## Discussion

Despite large R&D investments in preclinical studies only 4% of drug development pipelines yield licensed drugs [35] due to the low predictivity of such studies in terms of therapeutic efficacy and due to the fact that the definitive evidence of the validity of a new drug target for a disease is not validated until late phase development is completed. Therefore, the selection and prioritization of good drug targets is a central problem in drug discovery.

We presented a Machine Learning approach to prioritize proteins according to their similarity to approved drug targets. The main characteristic of our approach is the fact that it is completely unbiased. We computed a large collection of protein features and let the learning method score the features that best discriminate between approved targets from other proteins.

The interaction between drugs and their targets activates signaling cascades through Protein-Protein Interaction (PPI) networks causing downstream perturbations in the cell’s transcriptome. A (PPI) network models the cascade of relationships between targets and proteins by using physical contacts, genetic interactions, and functional relationships. Network analysis not only gives a systems-level understanding of drug action and disease complexity but can also help to improve the efficiency of drug design [36]. Hence various network measures (e.g., centrality measures, random walk, shortest path, nearest neighbor etc.) can be integrated with gene expression profiles to validate known drug targets or to identify essential proteins [20, 37]. Our results show, in a completely unbiased approach, that network centrality measures are among the most important discriminative properties to identify novel drug targets and characterize existing ones.

In addition to network centrality measures, other important features that our approach selects to score drug target are biological processes, essentiality and tissue specificity. Intuitively all these aspects are extremely important to characterize proteins associated with diseases. Interestingly the high importance score that the learning system gives to tissue specificity confirms the assumptions of large scale CRISPR-Cas9 screens adopted to prioritize cancer therapeutic targets and, surprisingly, our score is highly correlated to the one experimentally derived CRISPR-Cas9 data set [2].

The main bias toward cancer drug targets of our approach is related to the choice of the training set (102 approved targets) and the representation of the functional features associated with biological functions and signaling pathways, nevertheless our approach is general enough to be easily applied to other disease contexts with the appropriate selection of the training set and the corresponding representation of the functional features. We have shown that the method proposed here is highly predictive on a validation dataset consisting of 277 targets of clinical trial drug confirming that our druggability score is an efficient and inexpensive tool to guide the main choices in the process of drug discovery and development.

Finally, in order to apply an unbiased approach to score every possible protein, the method described here lacks of features based on genomic evidence [38, 39] as these features could not be extended to the whole proteome. Another important limitation of our method is that it is completely indirect, i.e. it does not take into account molecular models that use protein and drug structures to predict the binding between small molecules to the appropriate targets [40].

However, we believe that the target druggability score developed here is a complementary approach with respect to the ones based on genetic evidence and can be easily integrated with these more focused studies.

## Conclusions

We developed a machine learning approach to prioritize proteins according to their similarity to approved drug targets. We used the majority of features known to be differentiating drug targets from non-targets based on previous studies. We show that the network centrality measures are among the most unbiased predictive features to identify good drug targets and our results agree with those reported from large scale CRISPR-Cas9 screens. We made our predictions oncology specific by encoding the most cancer specific biological functions in our machine learning algorithm. This algorithm can be applied for other therapeutic areas, by combining the appropriate target training set with the most disease relevant gene ontology categories. Our method was highly predictive on a validation dataset consisting of clinical trial drug and recovered the vast majority of validated targets already targeted with many existing drugs. Our method also identified subset of novel proteins with high druggability scores but currently without any known drug targeting them.

## Methods

### Protein properties

The sequences of all human proteins were downloaded from the Uniprot database. Basic protein features were determined using the pepstat program of the European Molecular Biology Open Software Suite (EMBOSS) [41]. All properties generated from the Pepstat program were included in the prediction.

The Swiss-prot database was used to extract the post-translational modifications such as phosphorylation and glycosylation sites as well as the enzyme classification information.

### Tissue specificity

The RNAseq tissue expression data was downloaded from the Genotype-Tissue Expression (GTEx) and the Human Protein Atlas (HPA) [42, 43] databases. To determine the tissue specificity we calculated the entropy measure for a gene expression profile as described below. We note that Shannon entropy or similar measures have been used before to characterize tissue specificity of gene expression profiles [44]. The equation describes the tissue specificity of a gene (g), where *e*_*ig*_ is the gene’s expression in tissue *i*, N is the total number of tissues and S_g_ is the sum of expression in all tissues considered:

$$ E(g)=-\frac{1}{S_g}\sum \limits_{i=1}^N{e}_{ig}{\mathit{\log}}_2\frac{e_{ig}}{S_g} $$

The entropy of a gene’s expression ranges from zero for genes expressed in a single tissue to log_2_(*N*) for genes characterized by a uniform expression profile across all tissues. The entropy was calculated for both datasets separately and the averaged values were used for the predictions.

### Computational predictions of protein properties

In order to determine a complete set of features for all human proteins, we utilized a set of computational prediction methods. The input for all algorithms was the sequence of the proteins. We selected algorithms based on availability and feasibility in terms of computational times. A full list of the different methods utilized has been listed in Table 1.

**Table 1:** Computational methods to predict features

Name of the method	Predicted Feature	Reference website
NetPhos [45]	phosphorylation sites	http://www.cbs.dtu.dk/services/NetPhos/
GlycoMine [46]	glycosylation sites	http://glycomine.erc.monash.edu/Lab/GlycoMine/
WESA [47]	solvent accessibility	http://pipe.scs.fsu.edu/wesa/
Garnier [48]	secondary protein structure	http://www.bioinformatics.nl/cgi-bin/emboss/garnier
Epestfind [41]	PEST motif	http://emboss.bioinformatics.nl/cgi-bin/emboss/epestfind
SignalP [49]	signal peptide cleavage site	http://www.cbs.dtu.dk/services/SignalP/
CELLO [50]	cellular sub-localization	http://cello.life.nctu.edu.tw/
TMHMM [51]	presence of transmembrane helices	http://www.cbs.dtu.dk/services/TMHMM/

The list of computational methods utilized to predict target features.

### Statistical analysis and data normalization

Our protein features included continuous and categorical values. We applied the Wilcoxon Rank Sum and the Chi-squared test for the continuous and categorical variables, respectively as implemented in the R statistical software.

The determined features had a wide range of values and applying appropriate scaling was necessary. First, the features characterized by a heavy-tailed distribution (such as the network properties) were log-transformed. Second, all features were scaled to a number between zero and one by normalizing them to the difference between the maximum (*f*_*max*_) and the minimum of the feature (*f*_*min*_):

$$ {f}_{scaled}=\frac{f-{f}_{min}}{f_{max}-{f}_{min}}. $$

### Network properties

The protein interaction table was downloaded from the STRING database [24] and the top 10% of the highest scored interactions were used for the degree and centrality measure calculations. The degree, betweenness centrality, closeness centrality, pagerank and eigen centrality was calculated using the igraph R package.

### Identifying drug targets

We downloaded from the TTD website [6] the full database (version 6.1.01). This database contained 2917 unique protein targets. The downloaded data included 345 approved, 903 clinical trial and 1669 research targets. We filtered these targets based on the indications and kept only the ones with at least one indication related to oncology. Our final list of oncology targets consisted of 102 approved drug targets and an additional of 277 clinical trial drug targets.

We downloaded a second set of drug targets from the DrugBank database [5]. This data consisted of 2847 target protein with corresponding drug target information. We used this large dataset to add existing drug information to our list of prioritized targets.

### Gene ontologies

We used three ontology categories based on the content of Metabase: 1652 biological processes, 114 molecular functions and 246 signaling pathways. To determine the most important ontologies for cancer drug targets we performed an enrichment analysis of the 102 targets of approved drugs. The ontologies were rank-ordered based on their enrichment *p*-value (Fisher’s over-representation test). Ontologies that did not overlap with any of the approved targets were assigned a maximum rank value equal to the total number of ontologies in that category. Each protein was scored based on the rank of the ontology they belonged to. As in most cases, proteins were part of multiple ontologies we assigned the top 3 ranks to score them. Using this scoring system, the proteins characterized by cancer-specific functions were assigned top ranks resulting in large differences between the targets and non-targets (Figure S1).

### Predictions of target druggability score

The random forest models, predictions and performance evaluation were done using the “randomForest” and “caret” libraries in R statistical software. The training set was generated by combining the set of 102 approved oncology target drugs with a set of non-drug targets of the same size. The non-target set was defined as all proteins excluding targets with known approved drugs regardless of indications. This set of 18,604 non-drug target proteins was sampled without replacement to generate 102 non-drug targets for training the model.

The number of trees in each model was fixed at 1000 as any increase in trees above this value did not yield any significant changes in the prediction probability. We used the tuneRF function in the random forest package to determine the optimal number of variables (“mtry”) sampled at each split. The stepfactor was set at 0.01 and the “improve” parameter at 0.01 value. The predictions were averaged over 10,000 models generated by the random sampling of the non-drug targets (Fig. 2).

## Supplementary information

**Additional file 1: Figure S1.** The protein features differentiating between drug-targets and non drug-targets. The asterisk marks the significance level of the difference: *p* < 0.05 indicated as *, *p* < 0.01 indicated as ** and *p* < 0.001 indicated as ***. The red and black bars correspond to drug and non-drug targets, respectively.

**Additional file 2: Figure S2.** The protein feature correlation matrix plot.

**Additional file 3: Figure S3.** The drug target druggability score distributions for approved, clinical, withdrawn drugs and non-drug targets.

**Additional file 4: Table S1.** The list of approved drug targets in the training set and the list of clinical targets in the validation set.

**Additional file 5: Table S2.** The list of protein features utilized in the prediction model and the statistical significance differentiating the protein properties of drug targets from non-drug targets.

**Additional file 6: Table S3.** A. The prediction performance measures based on cross-validation as function of network interaction thresholds. B. Prediction performance based on the DrugBank targets. C. The prediction performance without network and gene ontology features.

**Additional file 7: Table S4.** The top 100 highest scored drug target and current drugs targeting them.

## Data Availability

The matrix of features for each protein is available at: https://figshare.com/s/b6c6566e7d73297bdd0c.

## Abbreviations

TTD: Therapeutic Target Database
PU: Positive Unlabeled
AUC: Area Under the Curve
TCGA: The Cancer Genome Atlas
DICE: Database of Immune Cell EQTLs
GTEx: Genotype-Tissue Expression
HPA: Human Protein Atlas
EMBOSS: European Molecular Biology Open Software Suite
CELLO: A subCELlular LOcalization
TMHMM: Transmembrane Helices Hidden Markov Models
WESA: Weighted Ensemble Solvent Accessibility
PPI: Protein-Protein interation
