# Supplementary material for: Machine learning prediction of oncology drug targets based on protein and network properties
Source: BMC Bioinformatics. 2020 Mar 14;21:104. doi: 10.1186/s12859-020-3442-9 (PMC7071582; doi:10.1186/s12859-020-3442-9)
Supplement: Supplementary file 1 — Additional file 1: Figure S1. The protein features differentiating between drug-targets and non drug-targets. The asterisk marks the significance level of the difference: p < 0.05 indicated as *, p < 0.01 indicated as ** and p < 0.001 indicated as ***. The red and black bars correspond to drug and non-drug targets, respectively. [file 12859_2020_3442_MOESM1_ESM.pdf]

Properties of drug targets

Subcellular localization

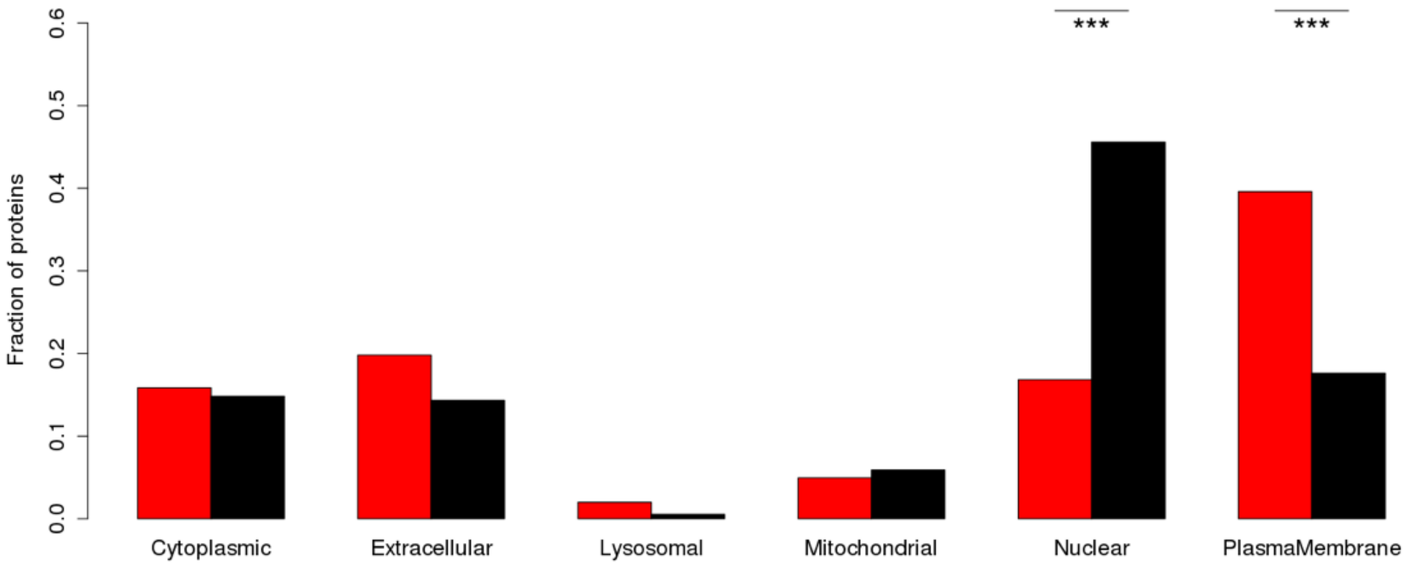

Protein essentiality

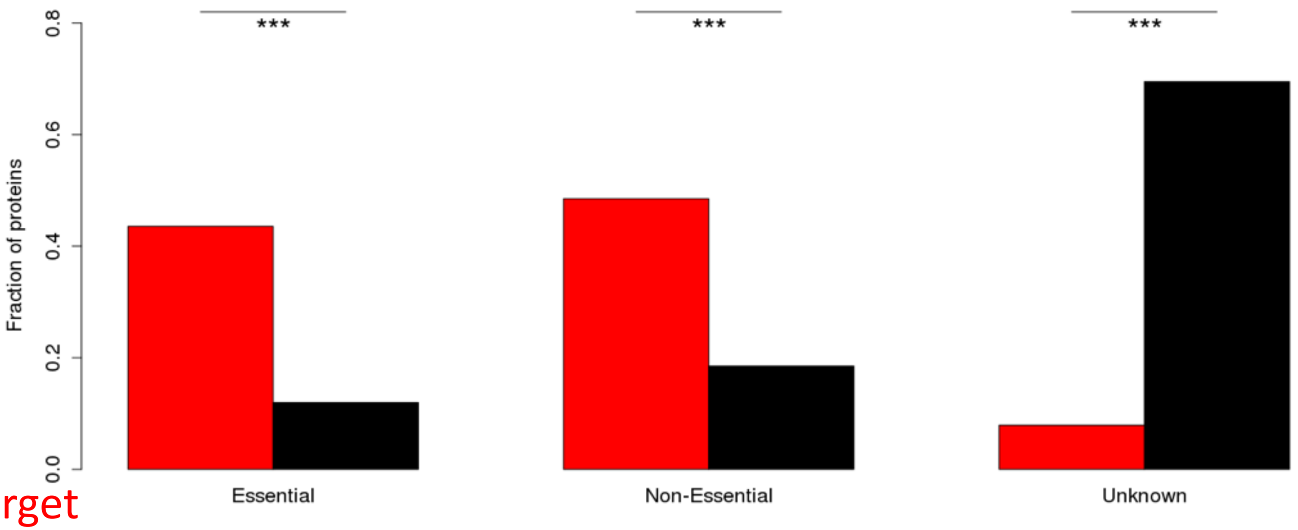

Red: drug target
Black: rest

Properties of drug targets

Enzyme classification

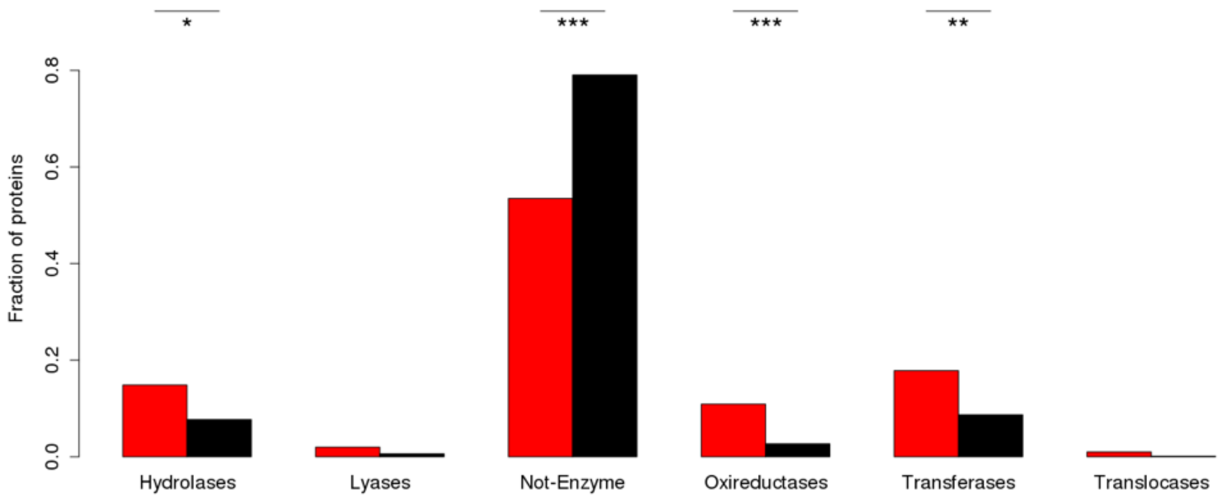

Post translational modifications

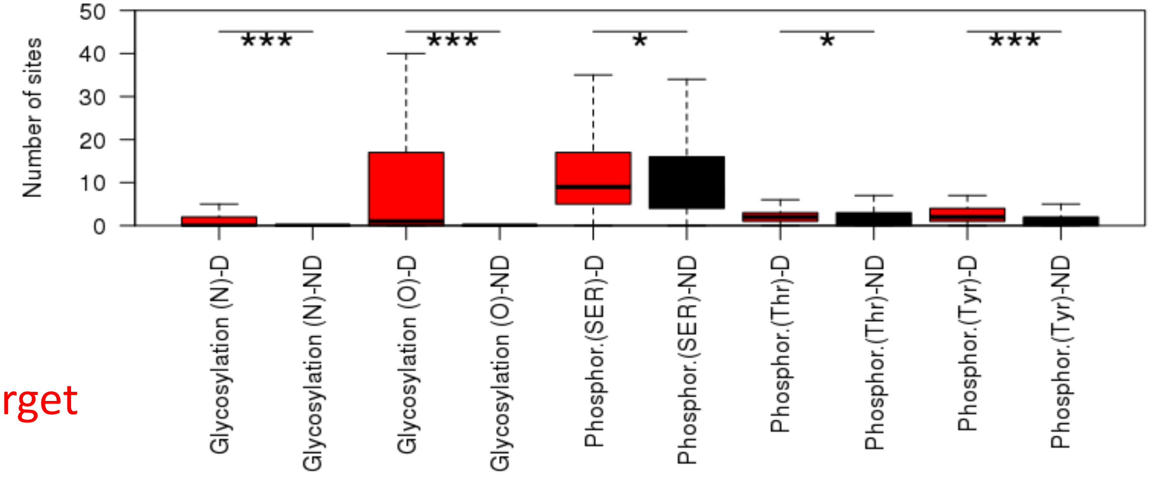

Red: drug target
Black: rest

Properties of drug targets

Transmembrane helices

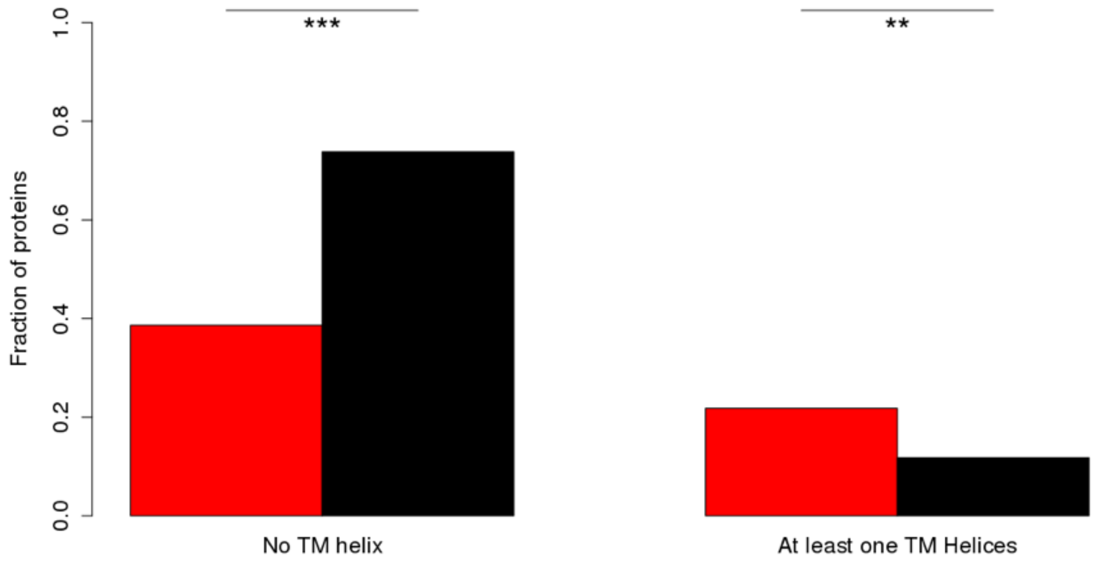

Signal peptide cleavage

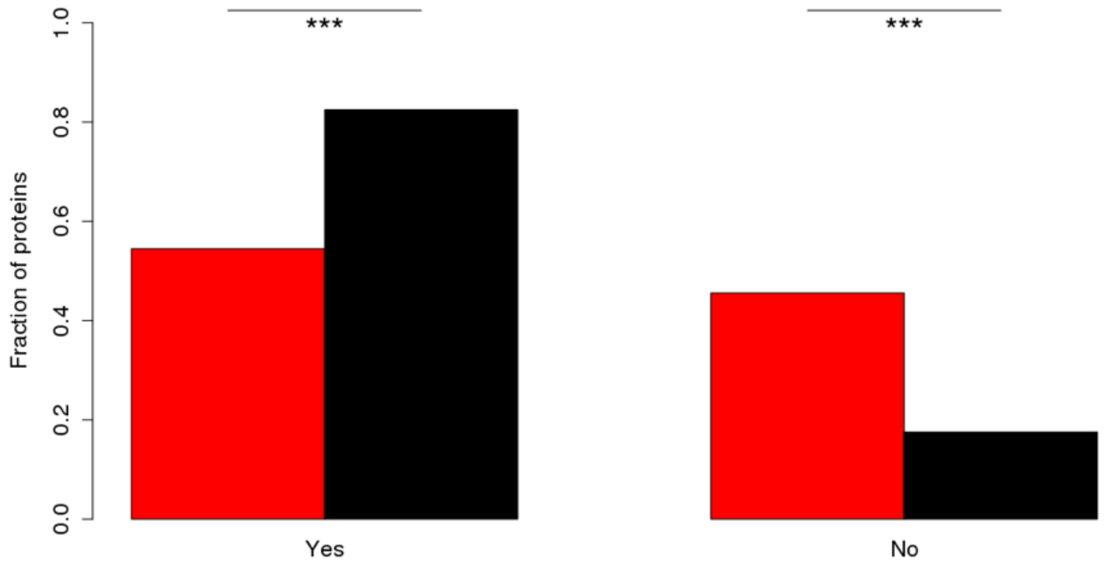

Red: drug target
Black: rest

Properties of drug targets

Secondary Structure

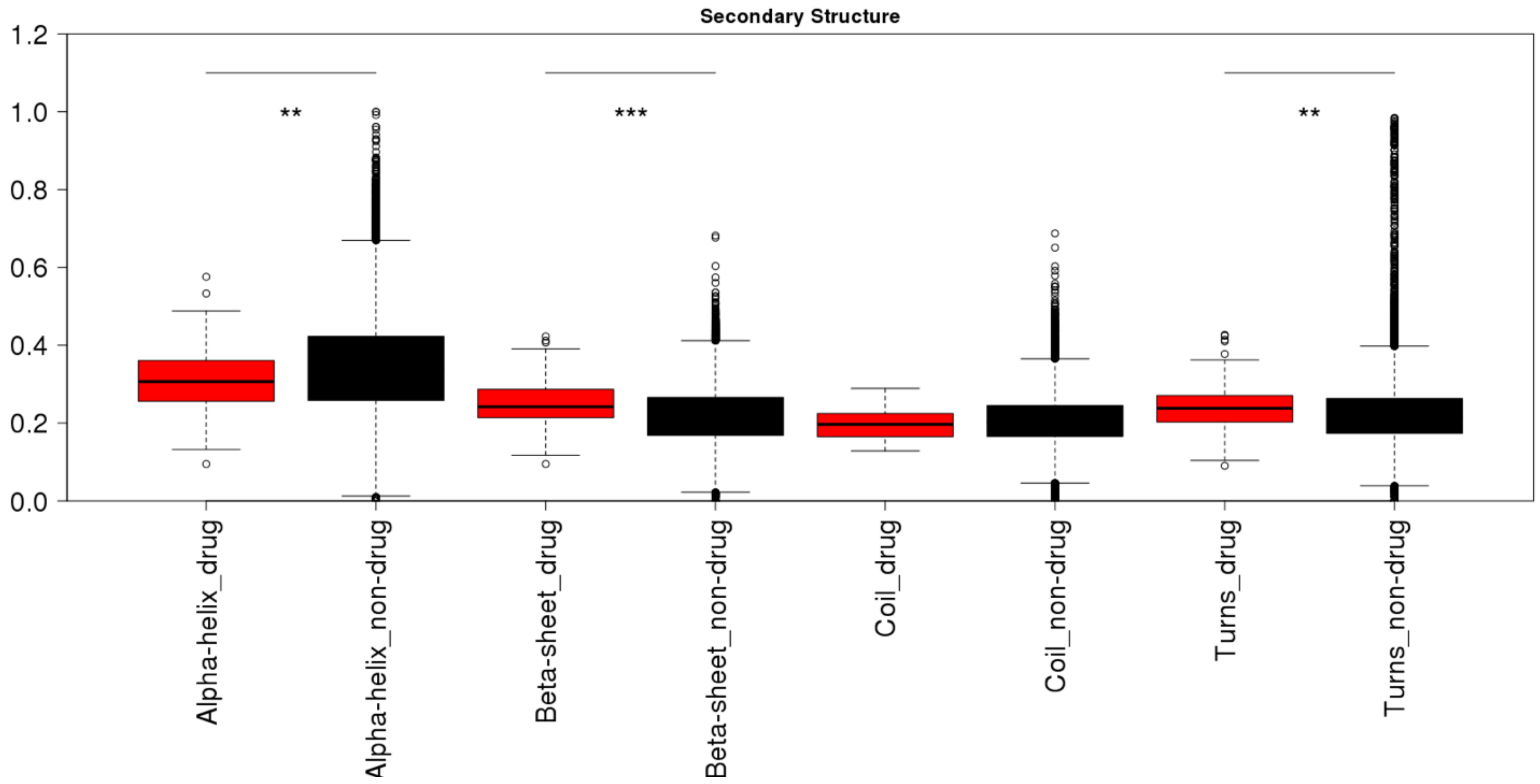

Red: drug target
Black: rest

Properties of drug targets

Physicochemical classes

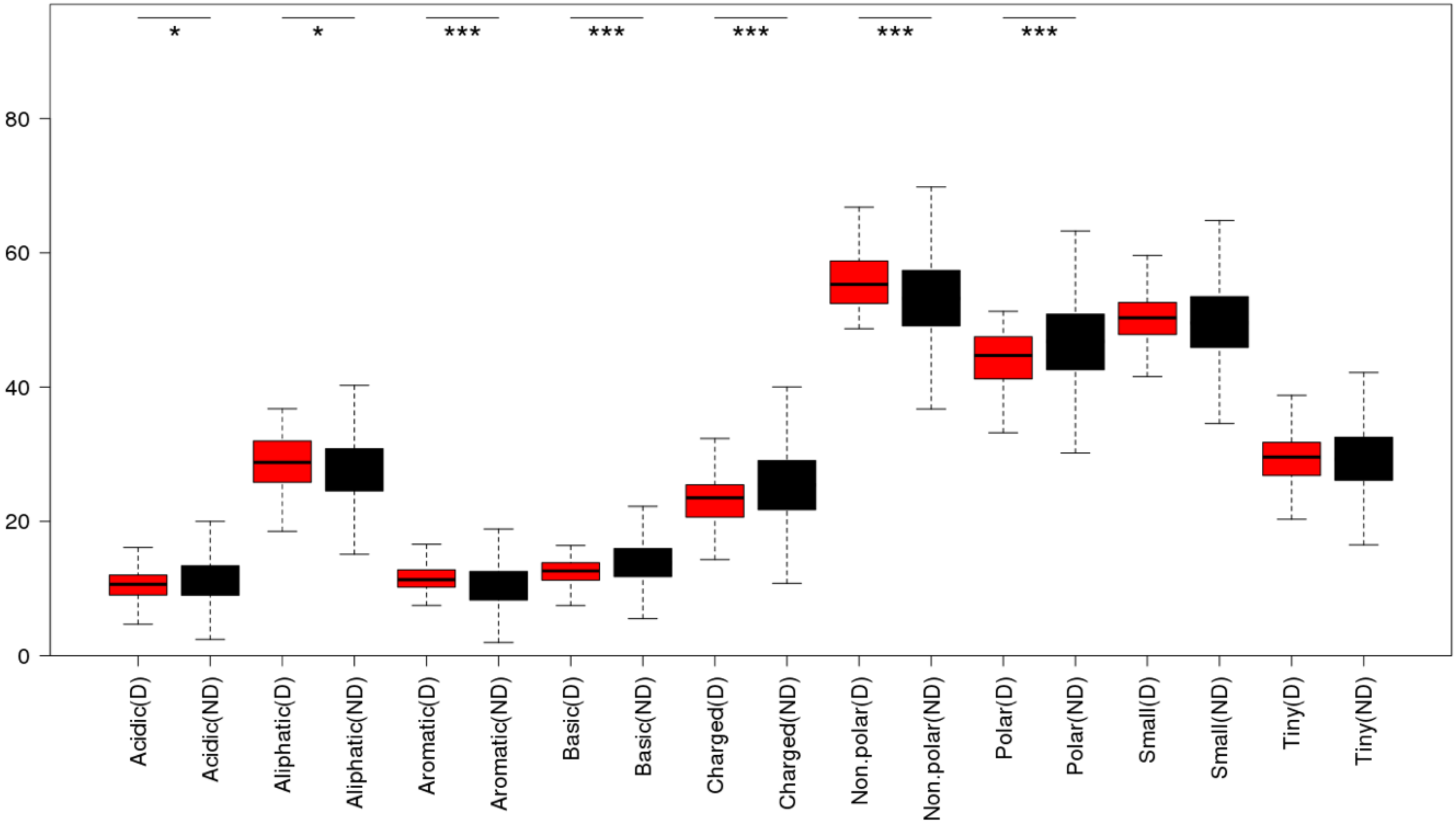

Red: drug target
Black: rest

Properties of drug targets

Protein sequence properties (normalized)

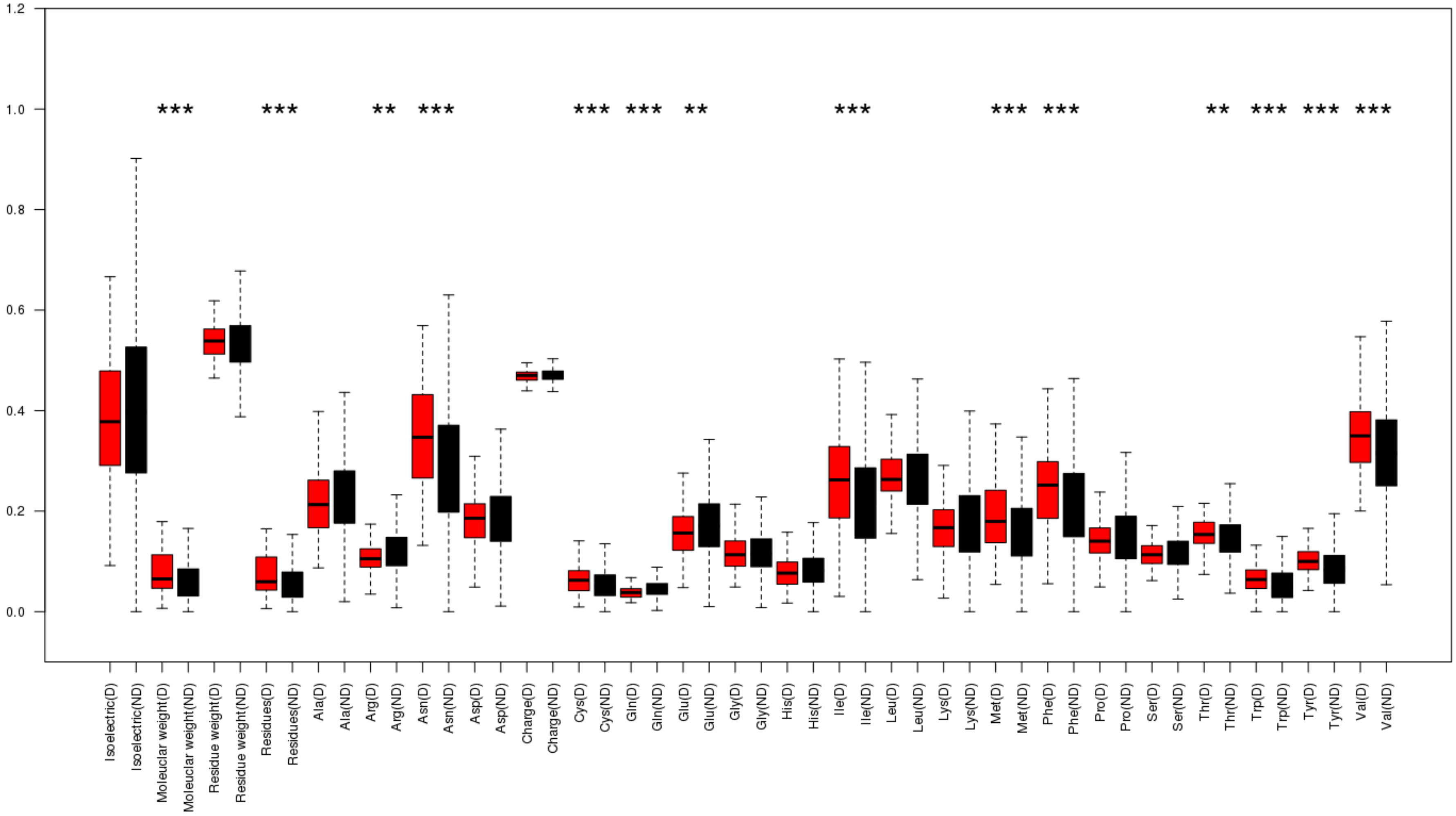

Red: drug target
Black: rest

Properties of drug targets

Tissue specificity

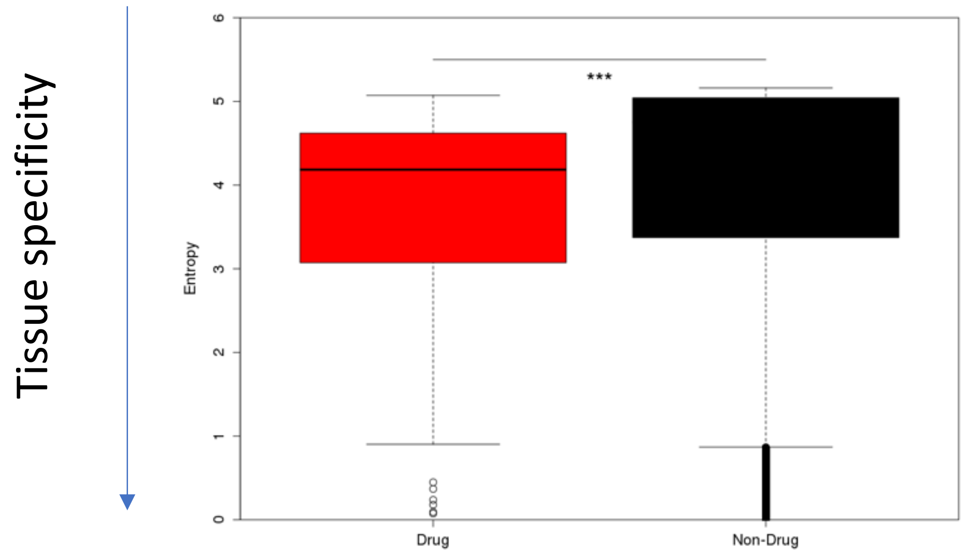

Solvent accessibility

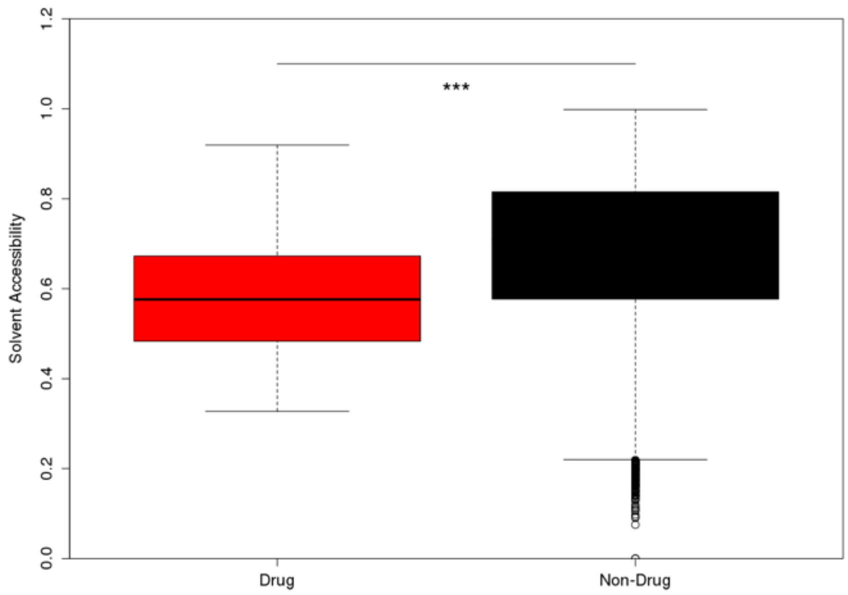

Red: drug target
Black: rest

Properties of drug targets

Network properties

Degree

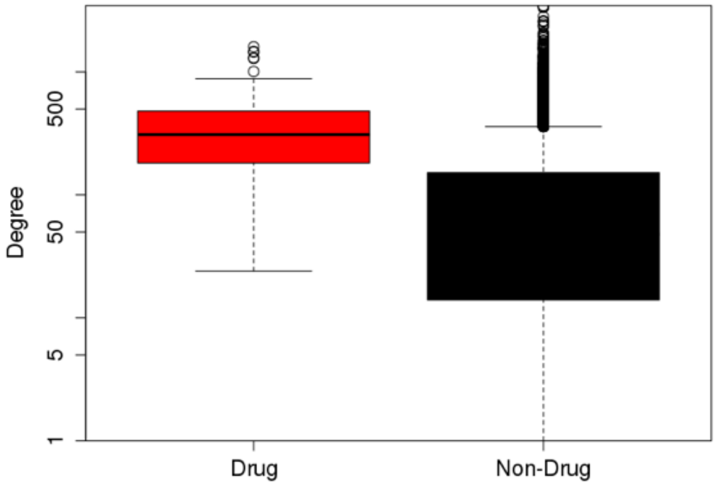

Betweenness

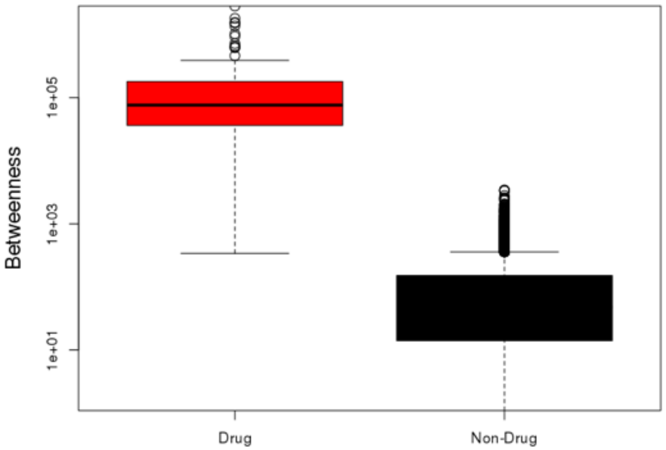

PageRank

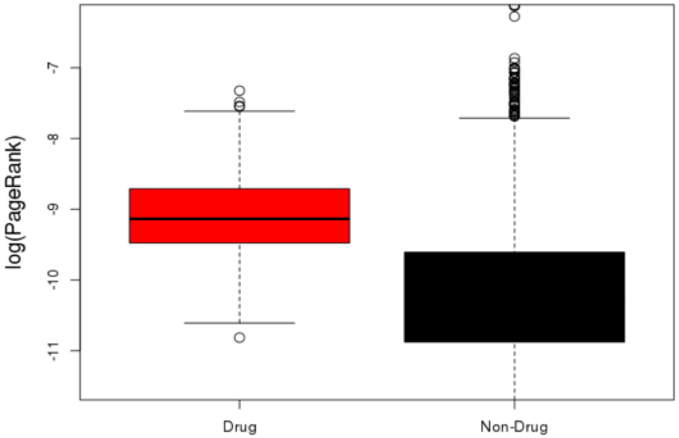

EigenCentrality

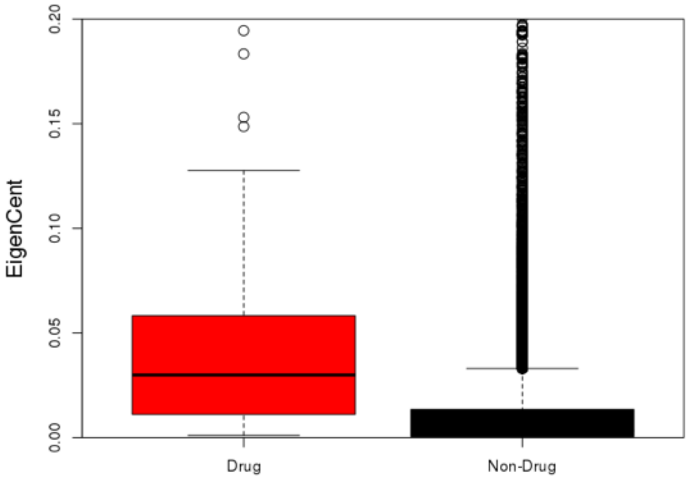

Red: drug target

Black: rest

Gene Ontology categories of drug targets

Biological Process Rank

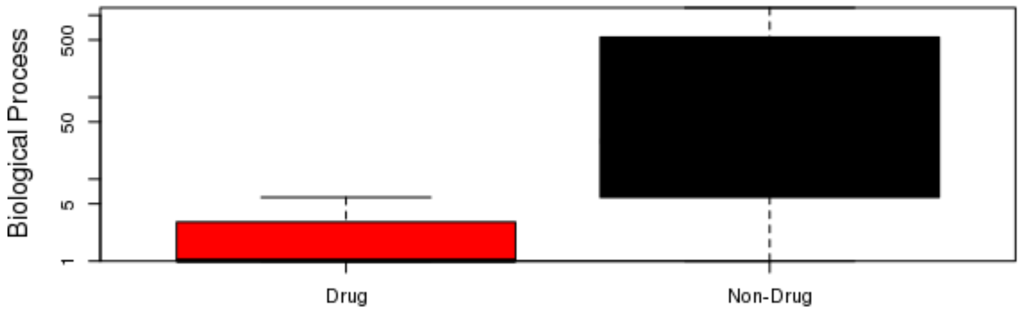

Molecular Functions Rank

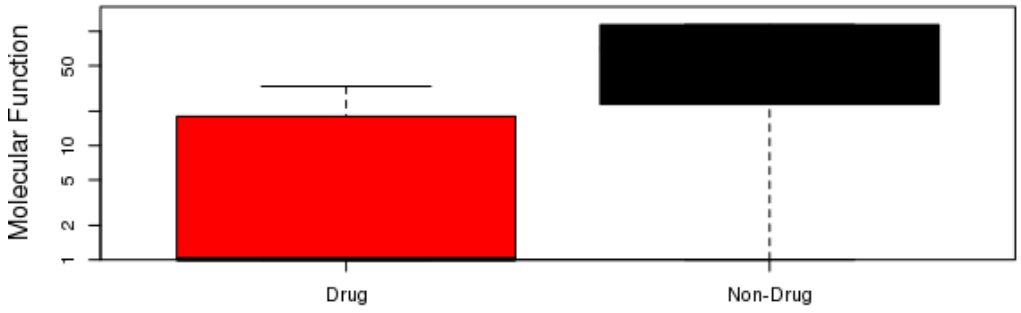

Pathway Maps Rank

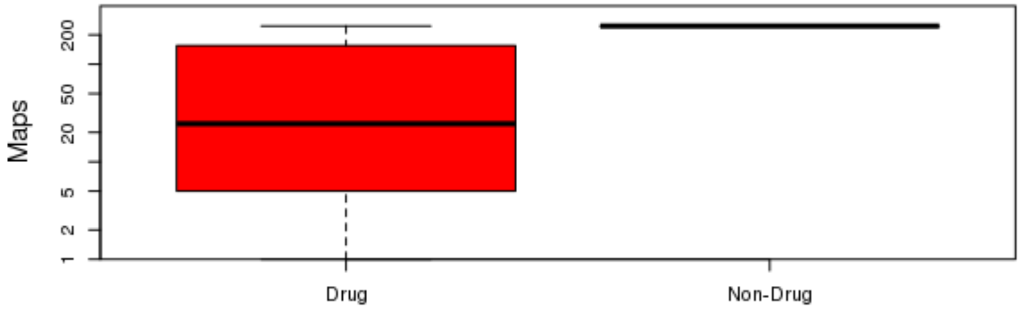

Red: drug target
Black: rest
