# Supplementary figures and images for: Machine learning prediction of oncology drug targets based on protein and network properties
Source: BMC Bioinformatics. 2020 Mar 14;21:104. doi: 10.1186/s12859-020-3442-9 (PMC7071582; doi:10.1186/s12859-020-3442-9)

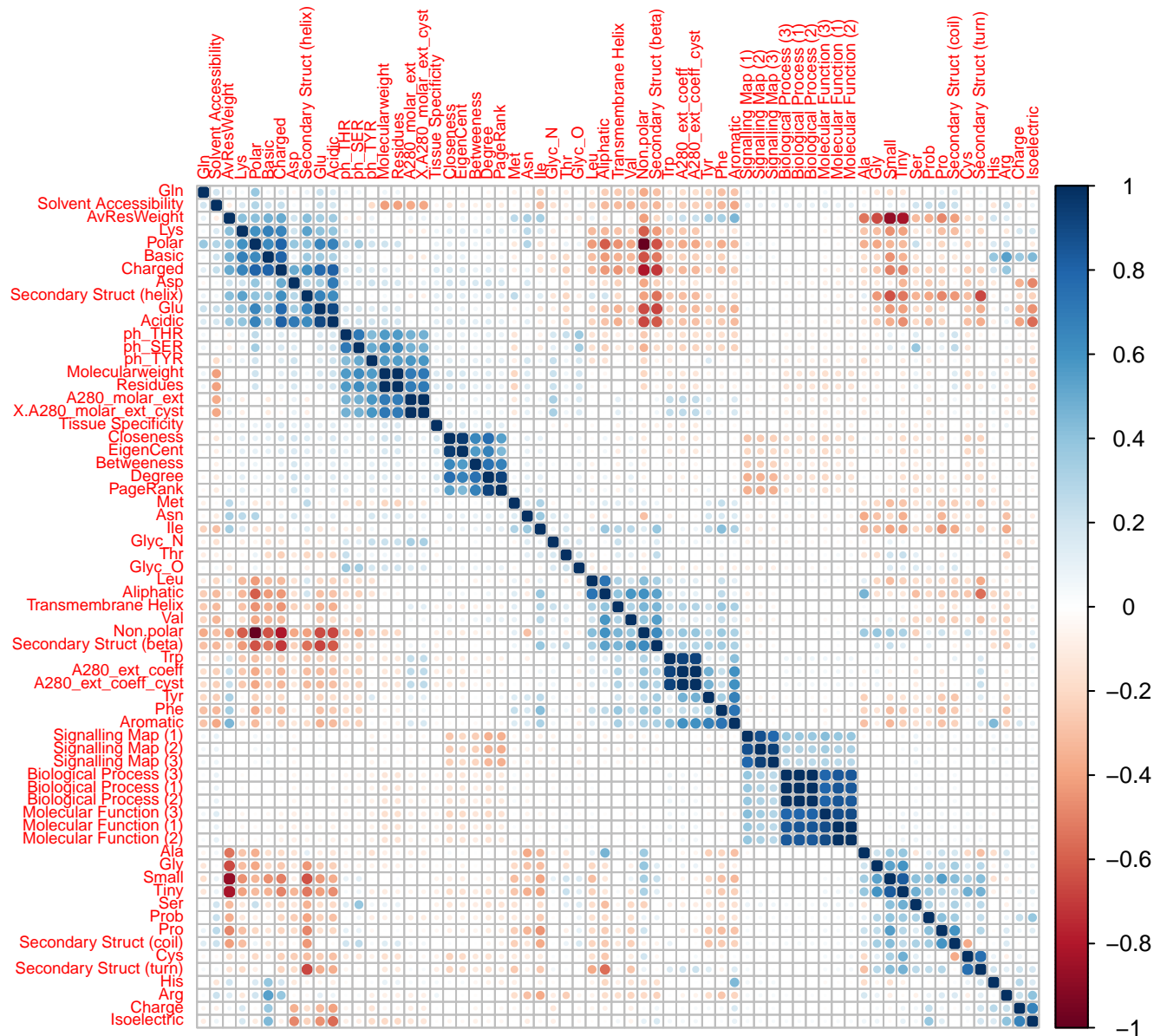

Supplement: Supplementary file 2 — Additional file 2: Figure S2. The protein feature correlation matrix plot. [file 12859_2020_3442_MOESM2_ESM.pdf]

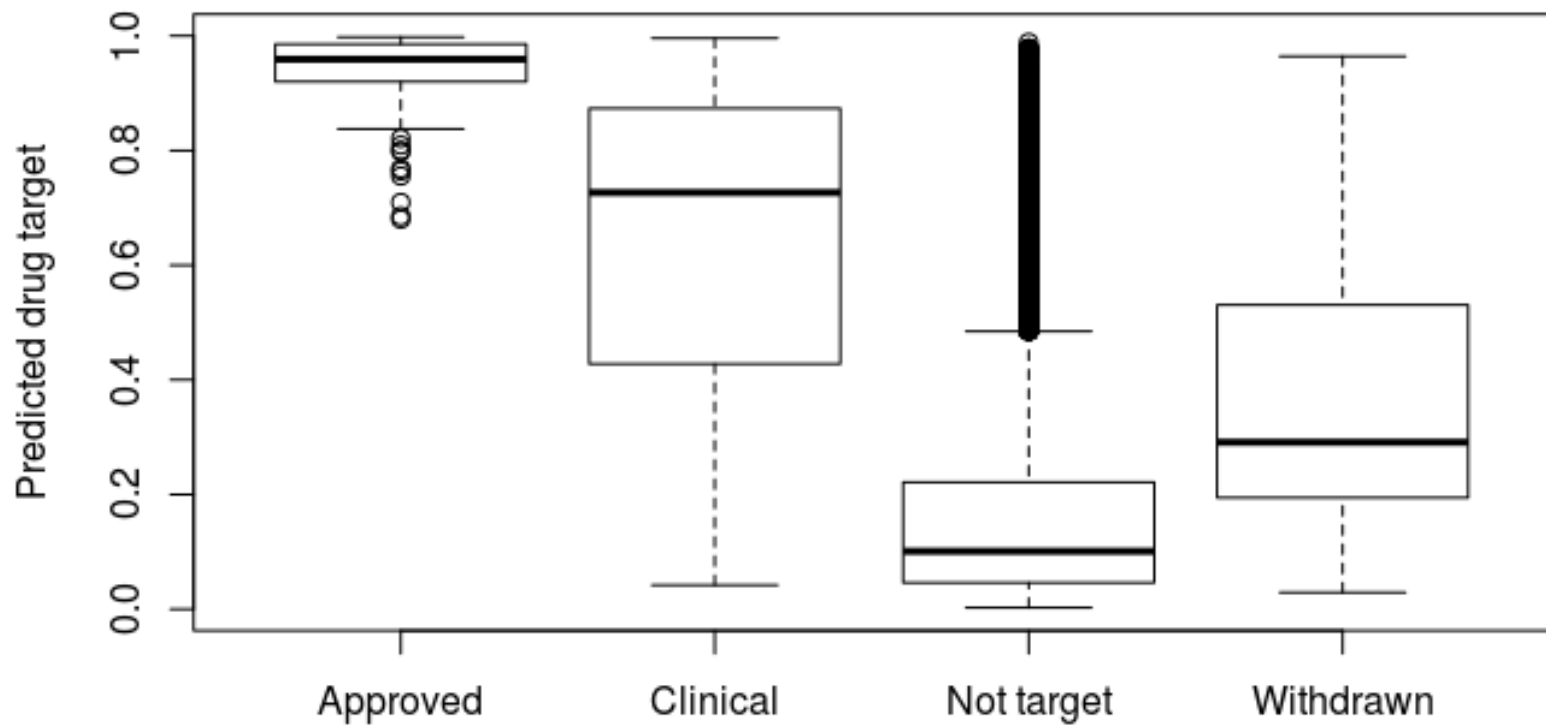

The prediction score differences between different sets of drug targets

Supplement: Supplementary file 3 — Additional file 3: Figure S3. The drug target druggability score distributions for approved, clinical, withdrawn drugs and non-drug targets. [file 12859_2020_3442_MOESM3_ESM.pdf]
